# Role of carbonyl sulfide in acute lung injury following limb ischemia/reperfusion in rats

**DOI:** 10.1186/s40001-017-0255-z

**Published:** 2017-03-28

**Authors:** Yan-Rui Zhao, Wen-Rui Lv, Jun-Lin Zhou

**Affiliations:** Department of Orthopedics, Beijing Chao Yang Hospital, Capital Medical University, Gong Ren Ti Yu Chang Nan Rd, Chaoyang District, Beijing, 100020 People’s Republic of China

**Keywords:** Carbonyl sulfide, Acute lung injury, Ischemia/reperfusion

## Abstract

**Objective:**

To investigate the effect of carbonyl sulfide (COS) on limb ischemia/reperfusion (I/R)-induced acute lung injury (ALI) and the associated mechanism in rats.

**Methods:**

ALI was induced by bilateral hind limb I/R in Sprague–Dawley (SD) rats. Sixty-four SD rats were randomly divided into the control group, I/R group, I/R + COS group, and I/R + AIR group. We observed the survival rate of the rats and the morphological changes of lung tissues, and we measured the change in the lung coefficient, the expression levels of the intercellular adhesion factor-1 (ICAM-1) protein in lung tissue, the expression of tumor necrosis factor (TNF)-α, interleukin (IL)-lβ, and interleukin (IL)-6 in both lung tissue and serum, and cell apoptosis.

**Results:**

Limb I/R caused significant lung tissue damage. The number of polymorphonuclear neutrophil in alveolar septa, the expression level of the ICAM-1 protein in lung tissue, the expression levels of TNF-α, IL-1, and IL-6 in lung tissue and serum, the lung coefficient, and cell apoptosis all increased. When a low dose of COS gas was administered prior to limb I/R, the variation of the above indicators was significantly reduced, while an increase in the dose of COS did not reduce the lung injury but rather increased the mortality rate.

**Conclusion:**

Carbonyl sulfide is another new gaseous signaling molecule, and a low dose of exogenous COS may play a protective role in I/R-induced ALI by acting as an anti-inflammatory agent by promoting the production of antioxidants and by inhibiting the expression of adhesion molecule proteins.

## Background

Limb ischemia is a common clinical pathological sign. Restoring blood circulation to the limb is necessary to save the body, but it may aggravate the local tissue ischemia/reperfusion (I/R) injury, cause systemic inflammatory response syndrome when serious, or even cause distant multiple organ dysfunction syndrome. The lung is a target organ that is easily affected, and damage due to I/R injury can cause acute lung injury (ALI) and acute respiratory distress syndrome (ARDS), with an extremely high mortality rate (25–40%) [1]. Limb I/R-induced ALI remains a common problem for clinical doctors in the twenty-first century. Therefore, determining how to protect the lung tissue from limb I/R injury has become an important research objective.

Since the 1980s, many researchers have discovered toxic gas molecules, such as nitric oxide (NO), carbon monoxide (CO), hydrogen sulfide (H_2_S), and sulfur dioxide (SO_2_), to be biological gas signaling molecules; these gases play a very important role in physiology and pathophysiology, thus creating a new category of the “gas signaling molecule family.” The discovery of the “gas signaling molecule family” facilitated the emergence of a new era of lung injury research [2]. Our team previously published research on gas signaling molecules [3–5]. Recently, we focused our research on sulfurous gases [6–8], finding that H_2_S and SO_2_ have a protective effect on ALI induced by I/R in rats through regulation of the production of inflammatory and anti-inflammatory cytokines in the lung and plasma. Interestingly, we found another sulfurous gas, carbonyl sulfide (COS), when researching H_2_S and SO_2_. COS is a colorless, toxic gas with a rotten egg smell. It is an air pollutant that is also naturally present in the atmosphere, water, soil, and plants [9]. A previous study [10] reported that COS has vasodilator activity, but the specific mechanism is not clear. Currently, few reports on COS exist in the literature, and we have not yet seen any research reports on the effects of COS on ALI. According to previous research experience, we speculated that COS may be a new type of gas signaling molecule. Therefore, we designed this study with the aim of determining how low doses of exogenous COS affect limb I/R-induced ALI in rats.

## Methods

### Materials

Carbonyl sulfide was purchased from the Baijiatuan Village Gas Supply Station (Beijing, China). The TUNEL kit was purchased from Beyotime (Jiangsu, China). TNF-α, IL-6, and IL-1β enzyme-linked immunosorbent assay (ELISA) kits were purchased from Dakewe Biotech Company (Shenzhen, China). Analytically pure Na_2_SO_3_ and NaHSO_3_ were purchased from Beijing KangPuhui Technology Company (Beijing, China). Anti-ICAM1 antibody [YN1/1.7.4] (FITC) was purchased from Abcam (Shanghai, China).

### Animal model of induced ALI

Pathogen-free adult male Sprague–Dawley (SD) rats (180–230 g) were used in the study and were provided by the Experimental Animal Center of Chinese Academy of Sciences (Beijing, China). The Animal Ethics Committee of the Capital Medical University of China approved the study design, and all experiments were conducted in accordance with the established guiding principles for animal research. The rats were housed at a controlled ambient temperature of 23 ± 2 °C with 50 ± 10% relative humidity under a 12-h light–dark cycle (lights on at 8:00 a.m. and off at 8:00 p.m.). Standard rat chow and water were provided ad libitum to all rats. Sixty-four adult male SD rats were randomly divided into the following eight groups with eight animals per group: control group, I/R group, I/R + 0.2 COS group, I/R + 0.5 COS group, I/R + 1.0 COS group, I/R + 0.4 AIR group, I/R + 1.0 AIR group, and I/R + 2.0 AIR group. Control group rats underwent the full surgical operation but without I/R. The I/R + COS groups were given different volumes of COS (COS:AIR is 1:1) and the I/R + AIR groups were given the same volumes of air intraperitoneally (ip) 20 min before reperfusion.

Limb I/R-induced ALI animal models were performed the same as previously described in detail [7, 11]. The SD rats were anesthetized via ip administration of sodium pentobarbital (40 mg/kg body weight). An additional one-third dose of sodium pentobarbital was given hourly to maintain anesthesia. The left external jugular vein and the right carotid artery were cannulated for drug and fluid administration (Ringer’s lactate, 2 ml/h) and blood sample collection, respectively. A rubber tourniquet was used to bind the double hind leg root to cause limb ischemia. After 4 h, the tourniquet was loosened to allow for reperfusion. Laser Doppler blood flow detection (PeriFlux 5001, Perimed, Sweden) was used to ensure limb ischemia and reperfusion. Two hours after the reperfusion, the rats were euthanized using an ip injection of a lethal dose of sodium pentobarbital (90 mg/kg). Blood samples were drawn from the right ventricles using heparinized syringes and were centrifuged (4000 rpm, 10 min, 0–4 °C). Thereafter, plasma was aspirated and stored at −80 °C for the subsequent measurement of cytokine expression (IL-1β, IL-6, and TNF-α). Lung tissue from each rat was weighed, and portions of the tissues were then stored at −80 °C for the subsequent measurement of cytokine expression (IL-1β, IL-6, and TNF-α). Other portions of the tissues were fixed in 70% ethanol for the subsequent measurement of intercellular adhesion factor-1 (ICAM-1); the remainder of the tissues was fixed in 4% (vol/vol) neutral formalin for the detection of lung injury via hematoxylin and eosin (HE) staining. Apoptotic cells in the paraffin sections were identified using the one-step TUNEL apoptosis assay kit.

### Histopathological analyses and lung coefficient

Lung tissue samples were fixed in 4% (vol/vol) neutral formalin and were dehydrated through a graded ethanol series. After being embedded in paraffin wax, tissue samples were sectioned (4–5 µm) and stained with HE for light microscopy examination.

$$ {\text{Lung coefficient}} = {\text{lung wet weight (g)}}/{\text{body weight (kg)}}. $$

We removed the lung tissue by cutting off the trachea between the fifth and sixth cartilage ring above the tracheal bifurcation; then, the tissue was dried and weighed.

### TUNEL

Lung tissue samples were fixed in 4% (Vol/Vol) neutral formalin and then embedded in paraffin. Apoptotic cells in the paraffin sections were identified using the one-step TUNEL apoptosis assay kit according to the manufacturer’s instructions. A double-staining technique was used. TUNEL staining (green fluorescence) was used to quantify apoptotic cell nuclei, and DAPI staining (blue fluorescence) was used to quantify the total cell nuclei, as described by Omura [12]. The stained samples were observed under fluorescence microscopy (OLYMPUS IX51, Tokyo, Japan) with 488-nm excitation and 530-nm emission wavelengths. The cells with green fluorescence were defined as apoptotic cells. The apoptotic index was calculated as the ratio of the number of TUNEL-positive cells to the total number of cells. Five visual fields were randomly selected for each specimen.

### Levels of IL-1β, IL-6, and TNF-α in plasma and lung tissue

At various time points, lung tissue samples were thawed, homogenized, and centrifuged. The liquid supernatants were collected to test the concentrations of IL-1β, IL-6, and TNF-α. Blood samples (4 ml per rat) from each group were collected in heparinized tubes through jugular vein catheterization and were then centrifuged at 3000 rpm for 5 min. Cytokine levels were assayed using a double antibody sandwich ELISA following the manufacturer’s instructions. Samples (100 µl) and IL-1β standards (2000, 1000, 500, 250, 125, 62.5, and 31.3 pg/ml) were added to the ELISA plate wells. Each was tested in duplicate. Anti-rat IL-1β biotin (50 µl) was added to each well of the plates and was reacted for 90 min at 37 °C. The plates were washed with washing buffer four times; then, they were dried by tapping them upside down on filter paper. After washing, 100 µl of Streptavidin-HRP was added to the wells for 30 min at 37 °C. The plates were washed again four times, and 100 µl of TMB substrate was added to each well with gentle shaking for 10 s. The mixture was incubated in the dark for 30 min at room temperature. The reaction was terminated by adding 100 µl of stop solution to each well, and the optical density (OD) value at 450 nm was measured using a Varioskan Flash plate reader (Thermo Scientific, USA). The standard curve of the OD value vs. the concentration of IL-1β was obtained. The sample data were then plotted along the standard curve, and the sample IL-1β concentration was determined. The concentrations of IL-6 and TNF-α were assessed in the same assay.

### Level of ICAM-1 in lung tissue

We used the flow cytometry method (FCM, BD FACSAria, USA) to assess the level of ICAM-1 in lung tissue. First, PBS was used to wash the lung tissue specimens that were fixed in 70% ethanol. Then, samples of a single cell were prepared using a rub net method. PBS was used to wash 1 × 10^6^/ml lung tissue cells twice; then, the cells were centrifuged at 1000 r/min for 2 min and carefully aspirated, and the supernatants were discarded. The anti-ICAM1 antibody containing FITC (1 µg for 10^6^ cells) was added to the cells and was incubated in a water bath at 37 °C for 30 min. Then, the cells were centrifuged, washed twice, and aspirated; the supernatants were then discarded, removing the uncombined fluorescent antibody. The cells were resuspended in 1 ml of PBS and were then analyzed with the FCM to determine the fluorescence index (FI) and assess the relative content of ICAM1 protein using the following formula:

$$ \begin{aligned} {\text{FI}} & = ({\text{Sample protein expression of average fluorescence intensity}} \\ & \quad - {\text{control sample average fluorescence intensity}}) \\ & \quad /{\text{control sample average fluorescence intensity}}. \\ \end{aligned} $$

### Statistical analysis

All data are presented as the mean ± standard deviation. Statistical significance was assessed with one-way ANOVA followed by Tukey’s test. Statistical significance was set at *p* < 0.05. All analyses were performed using SPSS 17.0 (Chicago, IL, USA).

## Results

### Histopathological analyses and lung coefficient

**Fig. 1:**
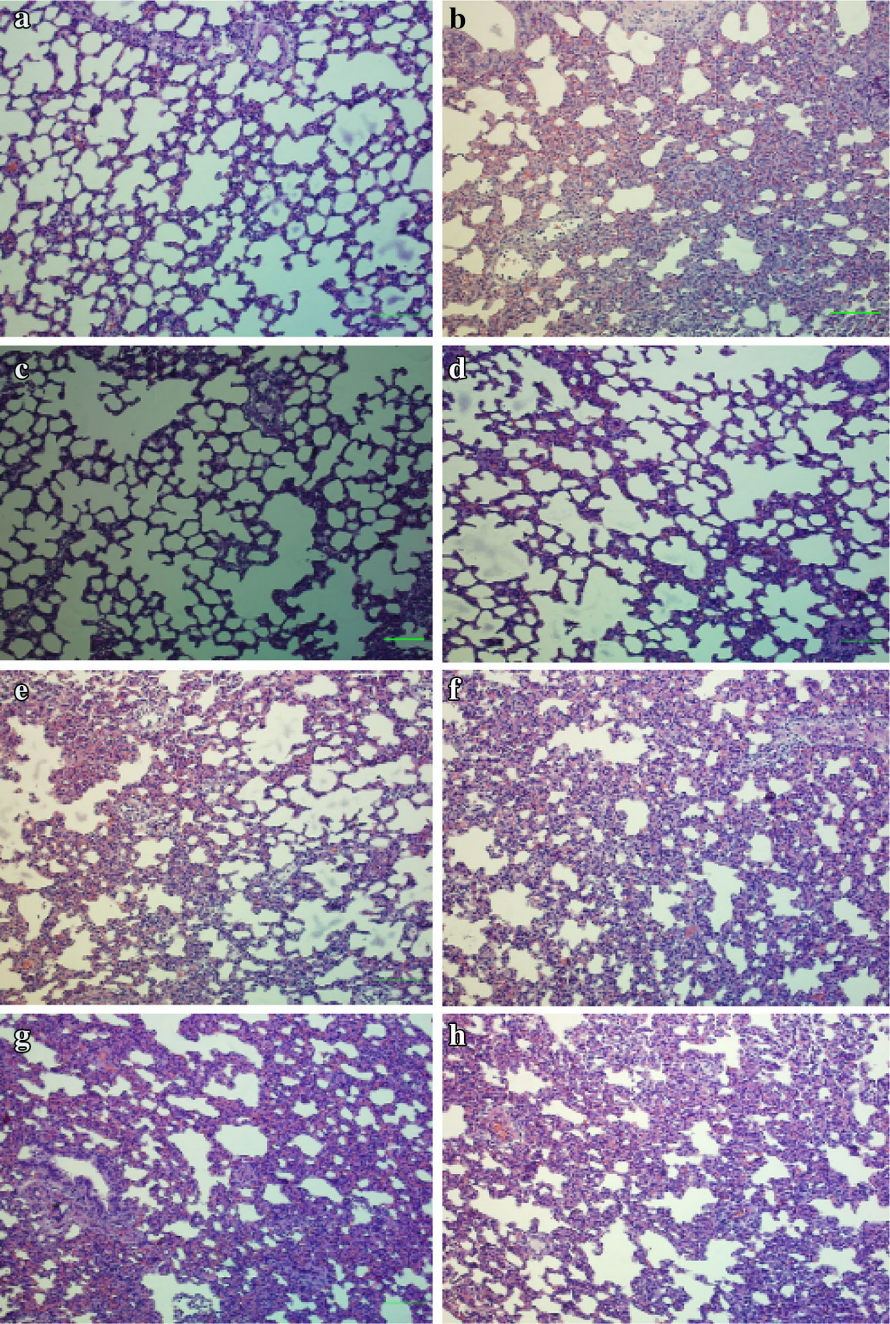
Lung morphological changes in limb I/R-induced ALI rats with different treatments. **a** Control group; **b** I/R group; **c** I/R + 0.2 COS group; **d** I/R + 0.5 COS group; **e** I/R + 1.0 COS group; **f** I/R + 0.4 AIR group; **g** I/R + 1.0 AIR group; **h** I/R + 2.0 AIR group

The morphological changes in the lung suggested the presence of inflammatory damage after 4 h of limb ischemia and then 2 h of reperfusion under the light microscope. The lung pathological sections from rats with ALI following limb I/R exhibited interstitial edema, alveolar thickening, and severe leukocyte infiltration in the interstitium and alveoli (Fig. 1b). The administration of COS attenuated the histological damage in the lung, especially in the I/R + 0.2 COS group (Fig. 1c–e). However, in the I/R + AIR group, little attenuation of lung damage in interstitial edema and leukocyte infiltration was observed (Fig. 1f–h).

Compared with the control group, the lung coefficient increased significantly in the I/R group (*p* < 0.01). Compared with the I/R group, the lung coefficient decreased in the I/R + COS group, with the I/R + 0.2 COS group exhibiting the most significant decrease (*p* < 0.01). Compared with the I/R + AIR group, the lung coefficient decreased significantly in the I/R + 0.2 COS and I/R + 0.5 COS groups (*p* < 0.01) (Table 1).

**Table 1:** The changes of lung coefficient, the contents of IL-1β, IL-6, and TNF-α in the plasma and lung tissue from limb I/R-induced ALI rats with different treatments

Group	LW/BW (g/kg)	IL-1β		IL-6		TNF-a	
		Blood	Lung	Blood	Lung	Blood	Lung
Control	4.91 ± 0.42	25.89 ± 3.66	29.13 ± 4.87	68.22 ± 3.45	63.76 ± 3.86	164.58 ± 11.57	166.13 ± 9.18
I/R	5.95 ± 0.45*	100.60 ± 4.55*	507.47 ± 42.88*	3161.75 ± 207.19*	3095.14 ± 133.31*	2140.42 ± 83.49*	2201.68 ± 110.98*
I/R + 0.2 COS	5.03 ± 0.34^#△^	66.97 ± 3.05^#△^	105.58 ± 7.00^#△^	448.89 ± 38.09^#△^	449.07 ± 35.90^#△^	1096.32 ± 68.80^#△^	1108.18 ± 83.42^#△^
I/R + 0.5 COS	5.1 ± 0.28^#△^	70.16 ± 6.81^#△^	126.77 ± 7.29^#△^	557.01 ± 46.16^#△^	543.93 ± 50.08^#△^	1273.14 ± 121.44^#△^	1274.66 ± 77.39^#△^
I/R + 1.0 COS	5.58 ± 0.26^#△^	77.95 ± 4.53^#△^	387.89 ± 16.94^#△^	2505.43 ± 310.50^#△^	2500.55 ± 252.13^#△^	1664.40 ± 46.01^#△^	1670.88 ± 46.86^#△^
I/R + 0.4 AIR	5.61 ± 0.29	100.30 ± 3.75	510.30 ± 17.02	3121.37 ± 298.35	3106.05 ± 169.37	2181.87 ± 128.33	2205.22 ± 156.34
I/R + 1.0 AIR	5.56 ± 0.34	100.96 ± 2.92	498.63 ± 20.41	2976.81 ± 261.15	3102.09 ± 218.91	2106.59 ± 137.97	2096.89 ± 166.49
I/R + 2.0 AIR	5.6 ± 0.39	98.66 ± 5.08	490.28 ± 16.71	3152.10 ± 220.3	3125.53 ± 153.9	2163.42 ± 164.04	2164.85 ± 118.42

Mean ± SD. *n* = 8

* *p* < 0.01 vs. control group, ^#^ *p* < 0.01 vs. I/R group, ^△^ *p* < 0.05 vs. I/R + AIR group

### Levels of IL-1β, IL-6, and TNF-α in plasma and lung tissue

To corroborate the histopathological and lung coefficient results, we tested the expression level of cytokines in the plasma and lung tissue using a double antibody sandwich ELISA. This technique enabled us to study the effects of COS on the expression of cytokines in lung injury. As shown in Table 1, compared with the control group, the concentrations of IL-1β, IL-6, and TNF-α in the plasma and lung tissue obviously increased in the I/R group (*p* < 0.01). Compared with the I/R group, the I/R + 0.2 COS and I/R + 0.5 COS groups exhibited significant decreases in the concentrations of IL-1β, IL-6, and TNF-α in both the plasma and lung tissue (*p* < 0.01); the concentrations in the I/R + 1.0 COS group only changed slightly, and those in the I/R + AIR (0.4, 1, 2 ml) group had minimal change (*p* > 0.05). Compared with the I/R + AIR group, the I/R + 0.2 COS and I/R + 0.5 COS groups exhibited a significant decrease in the concentrations of IL-1β, IL-6, and TNF-α in both the plasma and lung tissue (*p* < 0.01) (Table 1).

### TUNEL results

**Fig. 2:**
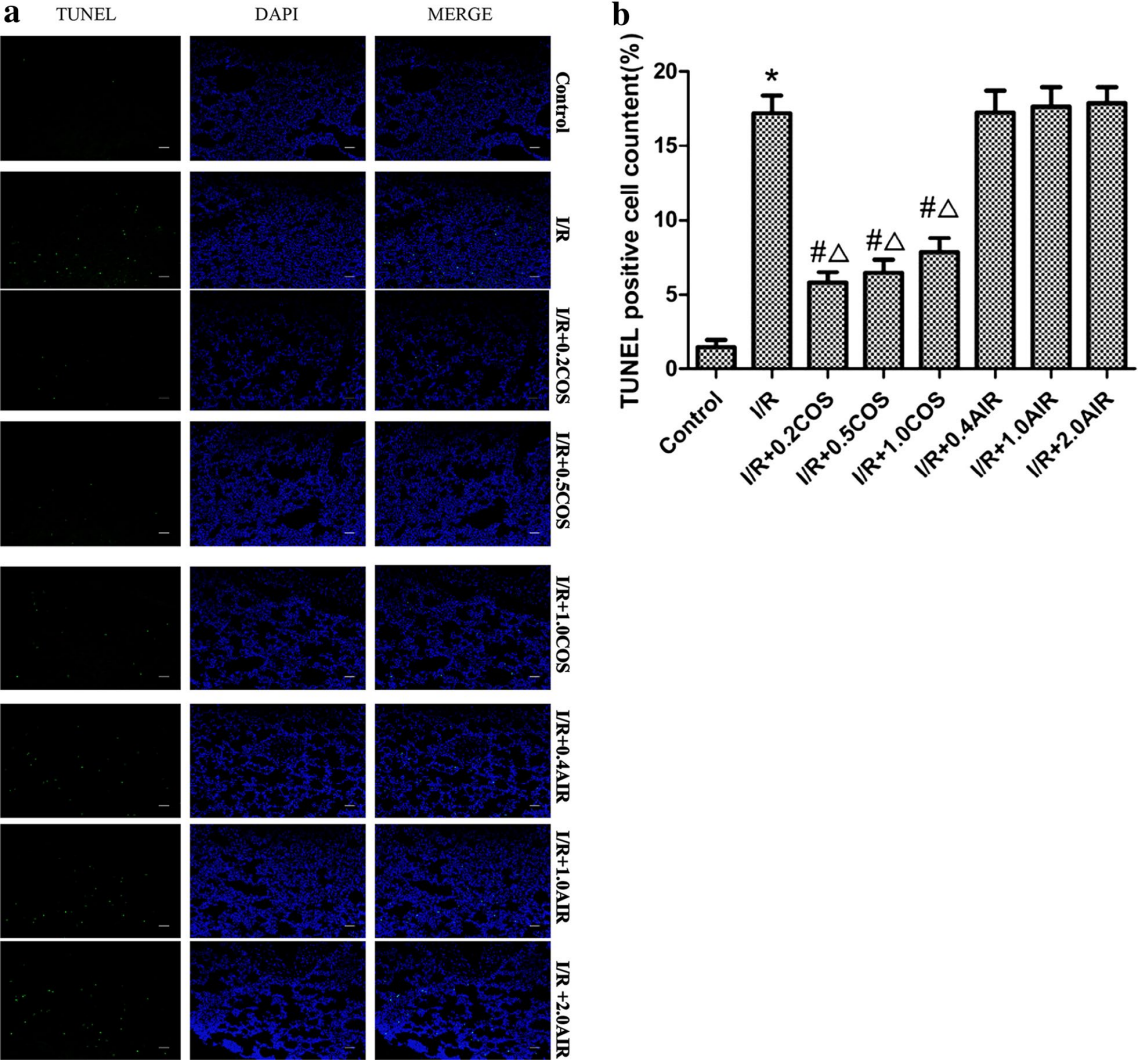
**a** Apoptosis analysis of the lung tissues in limb I/R-induced ALI rats with different treatments. DAPI staining and TUNEL assay. The lung tissue nuclei appeared light blue, TUNEL-positive nuclei appeared green (*n* = 8, 5 fields for each specimen). **b** The quantitative analysis of the apoptotic index in different experiment groups. Results are given as mean ± SD (*n* = 8 animals/group). Significant differences are indicated as: *p < 0.01 vs. control group, ^#^ *p* < 0.01 vs. I/R group, ^∆^ *p* < 0.05 vs. I/R + AIR group

To further evaluate the effects of COS on ALI, we assessed apoptosis using the one-step TUNEL apoptosis assay kit. As shown in Fig. 2, very few apoptotic cells were observed in the control group, and the apoptotic index was 1.47%. The percentage of apoptotic cells in the I/R group was obviously increased, and the apoptotic index increased to 17.2%. Compared with the I/R group, such an effect was significantly attenuated by adding different volumes of COS (0.2, 0.5, 1 ml), resulting in apoptotic indexes of 5.83, 6.48, and 7.88%, respectively. However, no change was observed in the number of apoptotic cells in the I/R + AIR (0.4, 1, 2 ml) group; their apoptotic indexes were 17.25, 17.65, and 17.85%, respectively (Fig. 2).

### Level of ICAM-1 in lung tissue

**Fig. 3:**
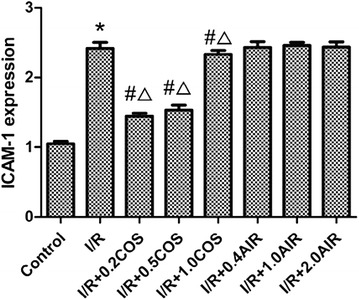
Effects of COS on ICAM-1 expression in the lung tissues. Results are given as mean ± SD (*n* = 8 animals/group). Significant differences are indicated as: *p < 0.01 vs. control group, ^#^ *p* < 0.01 vs. I/R group, ^∆^ *p* < 0.05 vs. I/R + AIR group

ICAM-1 is an important adhesion molecule on the surface of vascular endothelial cells that can promote leukocyte adhesion to endothelial cells when shock and inflammation occur, leading to clogged microcirculation and shock deterioration. To determine the effect of COS on the expression of ICAM-1 in ALI, we tested it using the flow cytometry method. As shown in Fig. 3, compared with the control group, the expression level of ICAM-1 protein increased significantly in the I/R group (*p* < 0.01). Compared with the I/R group, the expression level of ICAM-1 protein decreased in the I/R + COS group, with the most significant decrease found in the I/R + 0.2 COS group (*p* < 0.01). Compared with the I/R + AIR group, the expression level of ICAM-1 protein decreased significantly in the I/R + 0.2 COS and I/R + 0.5 COS groups (*p* < 0.01) (Fig. 3).

## Discussion

I/R is an important pathophysiological process in clinical practice and was first described by Jennings in 1960 [13]. It not only exists in different species and organs but is also involved in various pathological processes, such as multi-organ failure, heart failure, and shock [14, 15]. Prevention and treatment of I/R injury remain a challenge for the medical field. Numerous articles reported that gas signaling molecules play a very important role in physiology and pathophysiology. Our research group has been engaged in the study of the effect of small gas signaling molecules on I/R-induced ALI for many years [3–8]. Recently, we identified a new gas, carbonyl sulfide (COS).

Carbonyl sulfide was first described in 1841 [16]. It has low molecular weight and is a colorless air pollutant gas that originates from a variety of sources. It is naturally present in the atmosphere, water, soil, and plants [9]. COS has also been used as a fumigant [17, 18]. COS is the most abundant sulfur compound that is naturally present in the atmosphere. The average total worldwide release of COS into the atmosphere has been estimated to be approximately 3 million tons/year, with less than one-third of this amount related to human activity [19]. Additionally, COS can also be found in some foods, such as cheese and prepared vegetables from the cabbage family.

Carbonyl sulfide originates from the metabolism of thiocyanate by thiocyanate hydrolase; however, this enzyme is not present in eukaryotes [20]. Interestingly, in 2003, Balazy et al. [10] first reported that COS can be detected in both the porcine coronary artery (PCA) and cardiac muscle and has the effect of dilating arteries. They also found that stimulation of PCA with acetylcholine could cause an increase in the synthesis of COS within the coronary artery. This result suggested that muscarinic acetylcholine receptors (mAChRs) are involved in regulating COS synthesis because mAChRs are found in the coronary artery [21]. However, we cannot exclude the possibility that COS could be of bacterial origin [22]. Thiocyanate is found in the blood, milk, and saliva of mammals at concentrations of 30–830 IM [23, 24] and has been thought to have antimicrobial functionality. Cysteine, cystine, lanthionine, djenkolic acid, and isothiocyanates have been reported as biogenic precursors of COS [25]. However, little is known regarding the enzymology of the processes leading to COS production in biological systems.

Based on the above background, we conducted our study from another point of view, using low doses of exogenous COS before researching the effect of low-dose exogenous COS on I/R-induced ALI and exploring its mechanism. The results from this study showed that the number of PMN in alveolar septa, the number of apoptotic cells, the expression levels of TNF-α, IL-1, IL-6, and ICAM-1 protein, and the lung coefficient were all significantly increased after limb I/R. However, these changes were significantly reduced after using low-dose COS (0.2 ml). Therefore, we hypothesized that low doses of exogenous COS could play a protective role by acting as an anti-inflammatory agent, by promoting the production of antioxidants and inhibiting the expression of adhesion molecule proteins. However, increasing the dose of COS, which does not strengthen its protective effect, increased the animal mortality rate. In the 0.5 ml COS group, two rats died (mortality rate of 25%). In the 1.0 ml COS group, three rats died (mortality rate of 37.5%). Our previous study used three different doses of COS daily (0.2, 0.5, and 1.0 ml) via an ip injection consecutively for 7 days. The rats’ daily performance and survival rate were observed; subsequently, the pathology of lung tissue was assessed (data not shown). The results showed that the rats in the 0.2 ml COS group had no abnormalities; the mortality rate was 12.5% in the 0.5 ml COS group; and the mortality rate was 25% in the 1.0 ml COS group. Thus, this experiment also used three dosages of 0.2, 0.5, and 1.0 ml COS. However, in this experiment, the mortality rate increased to 37.5% in the 1.0 ml COS group. We hypothesized that the toxic effects of COS combined with the I/R injury increased the mortality rate of the 1.0 ml COS group.

In conclusion, we speculate that COS is a new gaseous signaling molecule in addition to NO, CO, H_2_S, and SO_2_. Additionally, a low dose of exogenous COS may play a protective role in I/R-induced ALI by acting as an anti-inflammatory agent, by promoting the production of antioxidants, and by inhibiting the expression of adhesion molecule proteins. However, we do not know which signal transduction pathways are involved in this process; this is a part of our ongoing work. Otherwise, only a few COS studies have been conducted, and many mechanisms of COS are still not clear, such as the enzymatic process of COS in the body, the impact of exogenous COS on the production of endogenous COS, the impact of the mechanism of anti-inflammatory cytokine expression, the toxicology of COS, and the relationships among COS, SO_2_, and H_2_S. Those aspects that are mentioned above may also play a role in ALI and thus deserve further study.

## Conclusion

We speculate that COS is a new gaseous signaling molecule in addition to NO, CO, H_2_S, and SO_2_. A low dose of exogenous COS may play a protective role in I/R-induced ALI by acting as an anti-inflammatory agent, by promoting the production of antioxidants, and by inhibiting the expression of adhesion molecule proteins. Many mechanisms of COS still need further study.

## Abbreviations

COS: carbonyl sulfide
I/R: ischemia–reperfusion
ALI: acute lung injury
PMN: polymorphonuclear neutrophil
ARDS: acute respiratory distress syndrome
NO: nitric oxide
CO: carbon monoxide
H_2_S: hydrogen sulfide
SO_2_: sulfur dioxide
ip: intraperitoneal
ICAM-1: intercellular adhesion factor-1
